# The Radiologist as a Gatekeeper in Chest Pain

**DOI:** 10.3390/ijerph18126677

**Published:** 2021-06-21

**Authors:** Silvia Pradella, Giulia Zantonelli, Giulia Grazzini, Diletta Cozzi, Ginevra Danti, Manlio Acquafresca, Vittorio Miele

**Affiliations:** 1 Department of Emergency Radiology, University Hospital Careggi, Largo Brambilla 3, 50134 Florence, Italy; giulia.zanto@gmail.com (G.Z.); grazzini.giulia@gmail.com (G.G.); dilettacozzi@gmail.com (D.C.); ginevra.danti@gmail.com (G.D.); manlioacquafresca@gmail.com (M.A.); vmiele@sirm.org (V.M.); 2 Italian Society of Medical and Interventional Radiology (SIRM), SIRM Foundation, 20122 Milan, Italy

**Keywords:** chest pain, cardiac computed tomography (CCT), acute coronary syndrome (ACS), coronary artery disease (CAD), acute myocardial infarction (AMI), CAD diagnosis

## Abstract

Chest pain is a symptom that can be found in life-threatening conditions such as acute coronary syndrome (ACS). Those patients requiring invasive coronary angiography treatment or surgery should be identified. Often the clinical setting and laboratory tests are not sufficient to rule out a coronary or aortic syndrome. Cardiac radiological imaging has evolved in recent years both in magnetic resonance (MR) and in computed tomography (CT). CT, in particular, due to its temporal and spatial resolution, the quickness of the examination, and the availability of scanners, is suitable for the evaluation of these patients. In particular, the latest-generation CT scanners allow the exclusion of diagnoses such as coronary artery disease and aortic pathology, thereby reducing the patient’s stay in hospital and safely selecting patients by distinguishing those who do not need further treatment from those who will need more- or less-invasive therapies. CT additionally reduces costs by improving long-term patient outcome. The limitations related to patient characteristics and those related to radiation exposure are weakening with the improvement of CT technology.

## 1. Introduction

Chest pain is the second leading cause for patients reporting to the emergency department (ED) [1,2,3,4]. Of these patients, only 10–20% are diagnosed with ACS, and only in one-third of cases, the patient has an acute myocardial infarction (AMI) [5,6].

The clinical presentation of ACS is typical only in a minority of cases; therefore, the diagnosis can be difficult [7]. However, even today, about 2–10% of patients with ACS are lost [8,9]. Furthermore, there are categories of weaker patients in which the problem is more relevant such as cancer patients or more recently patients with COVID-19 [10,11,12,13]. The diagnosis of ACS, understood as unstable angina or non-ST-elevation myocardial infarction (NSTEMI), is based on a careful evaluation of the symptoms, the electrocardiographic trace, and the increase of high-sensitivity troponin (present in heart injury) [6,14,15]. To these tests, we can add the echocardiographic evaluation, which in some cases shows a regional contractility deficit.

Failing to carefully identify patients with ACS can have major consequences, and patients with AMI who are mistakenly discharged from an ED have about twice the risk of mortality as that of those who are hospitalized [5,16,17]. As a result, the current guidelines recommend a period of observation with further diagnostic investigations for those patients who have symptoms related to ACS but who show no evidence of myocardial ischemia. This is estimated to cost about USD 5–10 billion per year [14,15].

### 1.1. Computed Tomography and Magnetic Resonance Imaging Benefits

If it is true that treatments such as invasive coronary angiography (ICA) make it possible to distinguish between AMI and non-coronary myocardial disease, does there exist a risk in this last case of subjecting patients to unnecessary ICA or aggressive antithrombotic and antiplatelet therapy [16,17,18,19]. Over-treatment of these patients as well as the increase of both the length of hospital stay and the number of complications leads to a rise in healthcare costs [18,20].

In view of the recognized high negative predictive value of the coronary CT, this test allows the exclusion of a coronary disease with certainty, and the patient can be safely discharged [21].The use of CT permits not only the reduction of hospitalization days but also a long-term cost saving, allowing a better stratification of the prognostic risk [22].

However, in selected cases, coronary angiography remains the indispensable tool to reach a definitive diagnosis, identifying the culprit coronary lesion [2,23]. As well as demonstrating coronary stenosis, invasive angiography allows immediate treatment through angioplasty and stenting.

Moreover, there are situations in which coronary angiography does not allow the correct diagnosis as the heart injury is not a consequence of a coronary disease.

There are conditions where additional cardiac imaging is required over coronary lumen evaluation. Both cardiac computed tomography (CCT) imaging and cardiac magnetic resonance imaging (CMR) can give complementary and additional information to angiography.

**Figure 1:**
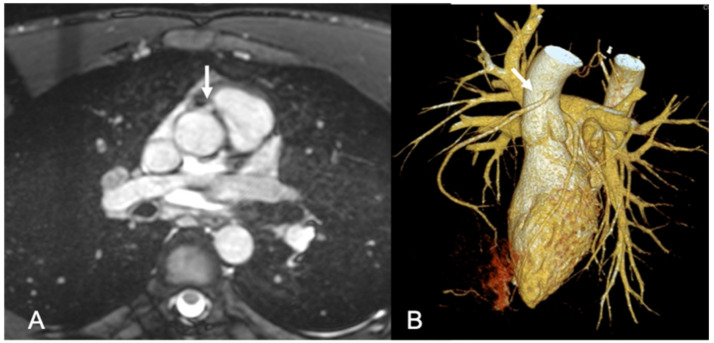
CMR performed for suspected cardiomyopathy in a 20-year-old male patient hospitalized for syncope and mild troponin elevation. A. In the whole-heart MR sequences without contrast agent, an anomalous origin of the right coronary artery (arrow) is present. The right coronary artery has a malignant course between the aorta and the trunk of the pulmonary artery (**A**). The CCT performed subsequently confirmed the coronary abnormality (**B**).

To date, magnetic resonance is not capable of adequately assessing coronary stenosis; on the other hand, it is possible to show the origin and course of the coronary arteries with this technique [1,22,23,24,25] (Figure 1).

**Figure 2:**
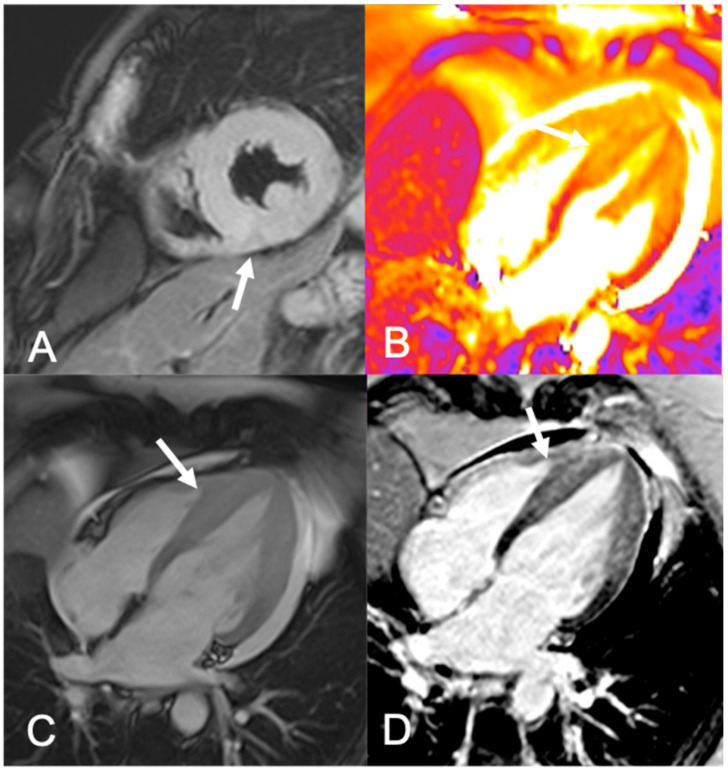
A 55-year-old woman on suspicion of Takotsubo syndrome. Coronarography showed no coronary stenosis. (**A**) T2-weighted short-axis image showing diffuse edema of the left ventricle middle–apical segments (arrow). (**B**) T2 mapping four-chamber view confirming the presence of edema (arrow). (**C**) Cine four-chamber TRUFI image showing thickening (arrow) of mid-apical segments of the left ventricle and hypokinesia. (**D**) In the four-chamber image, there was no appreciable ischemic pattern of LGE (arrow).

However, MRI has the unique ability to characterize the myocardial tissue and has the advantage of not using ionizing radiation. Cine sequences in cardiac planes allow the evaluation of ventricular function and volumes. Sequences for the evaluation of edema, late gadolinium enhancement (LGE), and mapping permit the highlighting of ischemic and non-ischemic alterations [24,25,26,27,28]. MRI can differentiate myocarditis from myocardial infarction with non-obstructive coronary arteries (MINOCA) or Takotsubo syndrome (TTS) in patients with troponin alterations who have undergone a coronary study by quantifying the edema and the pattern of myocardial damage [29,30,31] (Figure 2).

In addition, rest and stress CMR perfusion, in recent years, has shown an accurate assessment in various aspects of CAD both in diagnosis and in therapy [32]. In the European Society of Cardiology guidelines, the CMR is indicated as Class IA to prove myocardial ischemia before revascularization and perfusion. CMR is suitable for the assessment of symptomatic patients with intermediate (15–85%) pre-test probability of CAD [33,34]. The American College of Cardiology’s “appropriate use criteria” since 2014 consider perfusion CMR as an appropriate test in patients with intermediate pre-test probability and non-diagnostic exercise ECG as well as in patients with high pre-test probability regardless of the interpretability of the exercise ECG [32,35,36,37].

The CMR, however, is a test reserved for selected patients, this is due to the length of the procedure (up to about one hour) and the lower availability of scanners compared to that of those for CT [15].

### 1.2. Role of Coronary CT

Over the years, the role of coronary CT angiography (CCTA) has grown exponentially as it allows for plaque characterization and the visualization of the anatomy of the coronary and extra-cardiac structures [38,39,40]. Moreover, CCTA is able to detect other life-threatening non-cardiac causes of chest pain such as acute aortic dissection (AAS) and pulmonary embolism (PE) [1,40,41,42].

**Figure 3:**
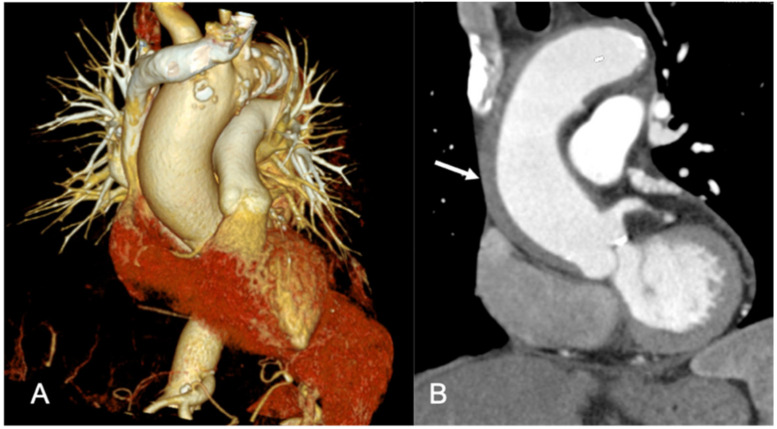
A 47-year-old woman with chest pain. (**A**,**B**) Combined CT evaluation of the aorta, pulmonary, and coronary arteries revealed type A dissection (arrow), no signs of pulmonary embolism or coronary stenosis.

Ideally, CCT angiography combined with thoracic angiography can precisely and quickly show the thoracic aorta, pulmonary arteries, and the coronary artery branches and also provide a full view of the lungs and mediastinum, which is an applicable exam for diagnosis in the case of acute chest pain [43] (Figure 3).

In clinical daily practice, with the most common CT scanners, aortic dissection and pulmonary embolism are usually excluded with CT angiography (without cardiac gated CT), and only if there is a specific suggestion, the study of coronary arteries is also performed [44].

Furthermore, with the new applications of “fractional flow reserve derived from CT” (CT-FFR) and “CT perfusion” (CTP), CCTA provides functional information [45,46].

**Figure 4:**
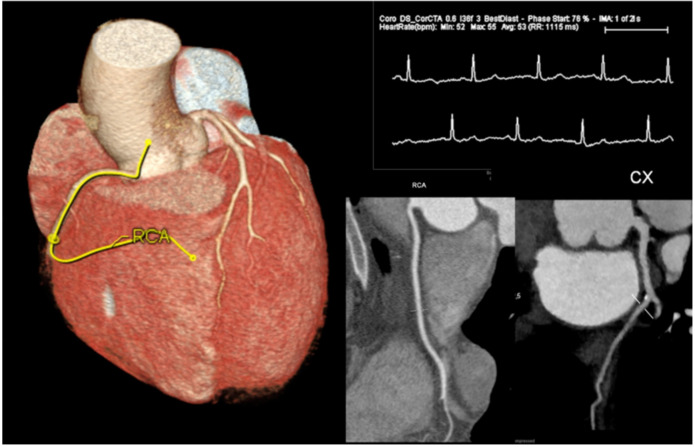
A 48-year-old male with intermediate risk of CAD and atypical chest pain. Cardiac gated CT showed no coronary stenosis, and the patient was safely discharged.

It is estimated that up to 10 million patients present with chest pain in the emergency room in the United States each year [2]. Only 10–20% are diagnosed with ACS and only in a third of cases, the patient has an AMI [47]. Failure to carefully identify these patients can have important consequences as patients with AMI mistakenly discharged from ED have about twice the risk of mortality as that of those who are hospitalized [48]. Even today, 2–10% of ACS cases remain undiagnosed [49,50]. In patients in whom ACS has not been excluded with certainty, the risk of cardiovascular events increases for at least five years; therefore, the efforts to get more accurate diagnostic tests are justified [51] (Figure 4). 

As opposed to ST-elevation myocardial infarction (STEMI), the electrocardiographic (ECG) setting can be normal in more than 30% of patients in non-ST-elevation ACS (NSTEMI) [5,52]. Measurement of a biomarker of cardiomyocyte injury, preferably hs-cTn, is mandatory in all patients with suspected NSTE-ACS [49,53]. According to the universal definition of acute MI, an increase in troponin with at least 1 value above the 99th percentile of a healthy population is suggestive of AMI [31].

However, it is often difficult to distinguish AMI from other disorders that result in elevated troponin levels, such as myocarditis, pulmonary embolism (PE), or Takotsubo cardiomyopathy (i.e., non-coronary myocardial disease) [50,54].

Invasive treatments such as coronary angiography (ICA) make it possible to distinguish between AMI and non-coronary myocardial disease, with the risk in the latter case of subjecting patients to unnecessary ICA or aggressive antithrombotic and antiplatelet therapy [1]. Over-treatment of these patients as well as increasing hospital days and the number of complications leads to an increase in healthcare costs [18,47].

Currently, the 2020 European guidelines of the European Society of Cardiology (ESC) lists as a class IA indication the use of CCTA as an alternative to ICA. Particularly, the ECR recommends CCTA to exclude ACS when there is a low-to-intermediate probability of CAD and when the cardiac troponin and/or ECG are normal or inconclusive [1]. Regarding the guidelines of the American College of Cardiology/American Heart Association (ACC/AHA) of 2012, the use of CCTA in patients with stable chest pain is indicated as Class IIb [3,55].

**Figure 5:**
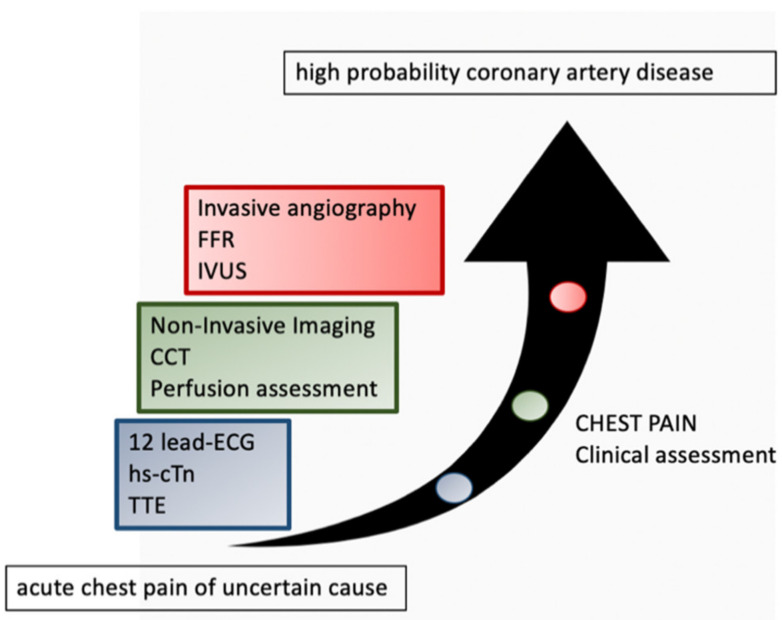
CCT angiography is thought to be a better gatekeeper and first-line test to triage patients and determine the need for medical therapy or invasive evaluation for those patients who are at low and intermediate risk of having CAD. FFR: fractional flow reserve; IVUS: intravascular ultrasound; CCT: coronary computed tomography; ECG: electrocardiogram; hs-CTn: high-sensitivity cardiac troponin; TTE: transthoracic echocardiogram.

Several studies tested CCTA against usual care in the management of patients presenting to emergency services with acute chest pain and low-to-intermediate risk of ACS (no signs of ECG ischemia and normal cardiac troponins) [6] (Figure 5).

Some limitations such as severe calcifications (high calcium score) and a high or irregular heartbeat still limit the use of the CCTA in selected cases [56]. Finally, the use of CCTA in the acute setting in patients with previous stents or coronary artery bypass grafting (CABG) has not been validated [1,40].

Over the years, the role of CCTA has grown exponentially as it allows for plaque characterization and the visualization of the anatomy of the coronary and extra-cardiac structures [54,57,58,59,60]. Furthermore, CCTA is able to detect other life-threatening non-cardiac causes of chest pain such as acute aortic dissection (AAS) and PE [6,15,38,61].

In large trials such as ROMICAT-II, 1370 patients with ACS were randomized to either a conventional treatment strategy or a second one involving coronary CT [59]. Patients in the CT group had a shorter duration of hospitalization at the PS (mean 18.0 vs. 24.8 h; *p* < 0.001). Furthermore, no deaths or infarcts were observed 30 days after the event in the group randomized to CT [59]. After 5 years, Reinhardt et al. carried out an analysis on secondary endpoints of the ROMICAT-II trial [39] and less-encouraging data emerged. Patients randomized to the conventional treatment strategy experienced a reduced incidence of diagnostic tests (*p* < 0.001) and coronary angiography compared to those randomized to CT (2% vs. 11%; *p* < 0.001), with lower costs in the first group. The remote evaluation, however, proved the use of the CT right; the use of a CT-driven protocol is beneficial in terms of outcome over standard assessment [62]. This result likely fits into the concept that better patient stratification leads to tailored therapies that lead to a better outcome [63].

Moreover, according to several clinical trials, CCTA can predict obstructive CAD better than traditional functional testing [17].

Several RCTs tested CCTA against usual care in the management of patients presenting to emergency services with acute chest pain and low-to-intermediate risk of ACS (no signs of ECG ischemia and normal cardiac troponins) [64]. At 1–6 months follow-up, there were no deaths; a meta-analysis demonstrated comparable results between the two approaches (i.e., no difference in the incidence of MI, post-discharge emergency room visits, or hospitalizations), further demonstrating that the CCTA was associated with a reduction of inward costs and length of stay in hospital [19].

However, none of these studies used hs-cTn tests, which also reduce hospital stay [53].

The VERDICT trial also proposes the use of coronary CT in this clinical context [16]. The study was designed to compare early invasive and selective invasive strategies in ACS patients. Overall, VERDICT included 2147 patients with positive troponin or ischemic electrocardiographic changes who were randomized to assess the effect of performing invasive coronary angiography either “very early” (within 12 h) or “standard” (within 2 to 3 days). Out of these 2147 patients, an additional coronary CTA examination prior to invasive angiography was performed in 1023 patients. The primary endpoint of the study was to evaluate the ability of coronary CT to rule out 50% stenosis, intended as a negative predictive value (NPV). Coronary CT NPV was 90.9% (95% CI: 86.8% to 94.1%); while the positive predictive value, sensitivity, and specificity were found to be 87.9% (95% CI: 85.3% to 90.1%), 96.5% (95% CI: 94.9% to 97.8%), and 72.4% (95% CI: 67.2% to 77.1%) [16,65]. The negative predictive value of over 90% allows for the exclusion of coronary heart disease with acceptable accuracy and allows the discharge of patients without significant stenosis on CT with reasonable safety.

The VERDICT study was carried out with an innovative technology (a 320-layer detector with reduced radiation dose) and a very different one from that used in previous years [16].

Over the years, technological advances have enhanced the capabilities of CCTA. Today’s latest scanners achieve a spatial resolution of up to 0.25 mm and temporal resolution of up to 40 ms, with sub-millisievert radiation doses [40,66,67,68,69].

However, some limitations such as severe calcifications (high calcium score) and a high or irregular heartbeat still limit the use of CCTA in selected cases. Finally, the use of CCTA in the acute setting in patients with previous stents or CABG has not been validated [1,8].

Obtaining high-quality, artifact-free images (among the most common: blooming, respiration, movement, cardiac pulsatility artifacts) in a selected patient is only part of the job of a cardiac imaging practitioner. A high level of competence is required to accurately interpret images and provide guidance for patient management and decision-making [40,70].

To standardize and facilitate the reporting of CAD on coronary CTA, in 2016, the Society of Cardiovascular Computed Tomography (SCCT), the American College of Radiology (ACR), and the North American Society for Cardiovascular Imaging (NASCI) established the Coronary Artery Disease Reporting and Data System (CAD-RADS) [71]. The CAD-RADS categories depend on the severity of the stenosis: the classification includes a range that goes from CAD-RADS 0 (absence of atherosclerosis) to CAD-RADS 5 (presence of at least one total occlusion) [71].

CAD-RADS 1 and 2 include non-obstructive CAD (degree of vessel stenosis less than 50%). No further evaluation is indicated in these categories [72].

Obstructive CAD is defined when the stricture affects more than 50% of the vessel lumen.

Moderate stenosis (50–69%) of at least one coronary artery needs functional evaluation to establish its hemodynamic effect, and the patient is assigned to the CAD-RADS 3.

CAD-RADS 4 category is divided into two subcategories: subcategory 4A includes severe stenosis (70–99%) of one or two coronary arteries, while subcategory 4B indicates left main artery stenosis >50% or obstructive disease of the three vessels (stenosis ≥ 70%) [71,73,74]. In patients with CAD-RADS 4A, a functional evaluation or ICA should be considered; in those with CAD-RADS 4B, invasive coronary angiography is recommended.

Complete occlusion (100% maximal coronary stenosis) of at least one of the vessels is classified as CAD-RADS 5, and ICA evaluation is required.

CAD-RADS categories can be supplemented by four modifiers that are added to specify whether a study is not fully evaluable or non-diagnostic (N) or to indicate the presence of stents (S) or grafts (G) and to report a vulnerable plaque (V). The “V” modifier should be added to the CAD-RADS category when an atherosclerotic lesion has two or more high-risk plaque features including positive remodeling, low attenuation plaque, spotty calcification, or napkin-ring sign [74].

Only coronary vessels with a diameter greater than 1.5 mm can be evaluated with CAD-RADS [72].

Preventive therapy and risk-factor modification are suggested for all patients with stable chest pain and category 1–5 CAD-RADS. Anti-ischemic drug therapy, hospitalization, and cardiac evaluation are recommended for CAD-RADS categories 3–5. Myocardial revascularization is recommended for CAD-RADS categories 4 and 5 [72].

The advancement of technology makes it possible to reduce the radiation exposure and the number of patients who cannot be evaluated with CT, making it an increasingly safe method [36,39,67,68,75].

This is a pathway that appears to be right. CT could become a gatekeeper for patients with acute coronary syndrome. The use of this approach would represent a clinical turning point, allowing a reduction in diagnosis times. To confirm the validity and effectiveness of this approach, however, further clinical studies are needed [63].

New CCTA applications in the areas of perfusion and fractional flow reserve are on the horizon and are set to expand the diagnostic utility of cardiac CT [36,76,77,78]. In addition, beyond the evaluation of the degree of coronary stenosis, CT is being proposed as a method for evaluating the morphologies, composition, and vulnerability of atherosclerotic plaques with new potential for using radiomics in this field and artificial intelligence to predict patient outcome [79,80,81,82].

## 2. Conclusions

Radiological cardiac imaging in recent years has evolved surprisingly, and CCT, in particular, plays a fundamental role today. Patients with chest pain who do not undergo invasive coronary angiography or surgery may benefit from this test to rule out the presence of coronary artery disease or even for coronary pathology stratification. CCTA is an applicable, safe, and fast modality for ruling out CAD in patients at low-to-intermediate risk presenting with acute chest pain. CCT permits to reduce hospitalization times, costs and, even more usefully, provides additional information regarding coronary disease that allows the customization of therapy with the aim of improving its outcome.
